# Continuous LED Lighting Enhances Yield and Nutritional Value of Four Genotypes of Brassicaceae Microgreens

**DOI:** 10.3390/plants11020176

**Published:** 2022-01-10

**Authors:** Tatjana G. Shibaeva, Elena G. Sherudilo, Alexandra A. Rubaeva, Alexander F. Titov

**Affiliations:** Institute of Biology, Karelian Research Center, Russian Academy of Science, 185910 Petrozavodsk, Russia; sherudil@krc.karelia.ru (E.G.S.); leksa_2018@mail.ru (A.A.R.); titov@krc.karelia.ru (A.F.T.)

**Keywords:** continuous lighting, photoperiod, spectral quality, light-emitting diodes, microgreens, functional food

## Abstract

The effect of continuous lighting (CL, 24 h) and light spectrum on growth and nutritional quality of arugula (*Eruca sativa*), broccoli (*Brassica oleracea* var. *italic*), mizuna *(Brassica rapa.* var. *nipposinica*), and radish (*Raphanus sativus* var. *radicula*) were investigated in growth chambers under light-emitting diode (LED) and fluorescent lighting. Microgreens were grown under four combinations of two photoperiods (16 h and 24 h) providing daily light integral (DLI) of 15.6 and 23.3 mol m^−2^ day^−1^, correspondingly) with two light spectra: LED lamps and fluorescent lamps (FLU). The results show that fresh and dry weights as well as leaf mass per area and robust index of harvested arugula, broccoli, mizuna, and radish seedlings were significantly higher under CL compared to 16 h photoperiod regardless of light quality. There were no visible signs of leaf photodamage. In all CL-treated plants higher chlorophyll *a/b* and carotenoid-to-chlorophyll ratios were observed in all plants except mizuna. CL treatment was beneficial for anthocyanin, flavonoid, and proline accumulation. Higher activities of antioxidant enzymes (catalase, superoxide dismutase, ascorbate peroxidase, and guaiacol peroxidase) were also observed in CL-treated plants. In most cases, the effects were more pronounced under LED lighting. These results indicate that plants under mild oxidative stress induced by CL accumulated more non-enzymatic antioxidants and increased the activities of antioxidant enzymes. This added nutritional value to microgreens that are used as functional foods providing health benefits. We suggest that for arugula, broccoli, mizuna, and radish, an LED CL production strategy is possible and can have economic and nutritional benefits.

## 1. Introduction

Microgreens are a relatively new specialty crop and emerging commodity in worldwide markets. They are defined as tender immature greens produced from seeds of vegetables, herbs, or gains, including local varieties and wild species having fully developed cotyledons with or without the emergence of a rudimentary pair of first true leaves [1,2]. Microgreen production is attractive to growers due to the increasing demand and high market value [3]. Microgreens are mainly used by chefs and consumers to enhance the color, flavor, texture, and nutritional value of various foods. Microgreens have a limited postharvest shelf life; therefore, local production, especially for fresh-cut products, represents the most promising production strategy [4]. Several species of microgreens are known for their health beneficial effects as they contain high concentrations of health-promoting phytochemicals [1,5]. Their bioactives include higher levels of antioxidant compounds such as polyphenols, carotenoids, and ascorbic acid, than their mature plants, thus qualifying microgreens as functional food [1,6]. 

Nowadays, growers produce microgreens in greenhouses and in plant factories with artificial lighting [7,8]. Indoor vertical farming is an environmentally sustainable form of plant production because of its high land and water resource use efficiency [8,9,10]. In controlled environment agriculture, such as greenhouses and plant factories with artificial lighting (PFAL), natural sunlight is supplemented or replaced with electrical lighting. Generally, lighting cost directly leads to higher production cost as it accounts for 70–80% of the total electricity consumption in PFAL [11]. Thus, it is necessary to reduce lighting cost through improving the lighting system with well-designed photosynthetic photon flux density (PPFD), photoperiod, spectrum, and control strategy. Although fluorescent (FLU) lamps were initially used as the standard light source in multilayer vertical growing systems, growers have begun replacing them with light-emitting diodes (LED). Compared to other artificial light sources, LEDs offer many advantages including low radiant heat output, low energy requirement, very fast response time, tunability, and long lifespan [12,13]. The availability of a large variety of narrowband-emitting diodes may allow for the scheduled induction of spectral-dependent physiological responses of plants, ensuring the optimal light setting for both crop yield and quality [14]. LED technology has shown a great potential to stimulate plant growth and the synthesis of bioactive compounds beneficial to human health [15,16]. With the advent of LED lighting fixtures and programmable control systems, the timing and intensity of horticultural lighting can be controlled more precisely than ever before. However, the selection of optimal lighting conditions for the growth of different plant species is far from resolved. Very little attention has been paid to the role of light quality in phytonutrient content of dietary plants [16]. Extensive literature data highlight varying responses of plants to different light settings even when same light conditions are applied to different species [17]. Therefore, an evaluation of the plant response to the light environment should be performed for each species as well as the growing conditions if the target is to maximize efficiency, yield, and quality of plants. 

As artificial lighting for growing plants is expensive, the solutions should be aimed to increase the efficiency of energy conversion into yield and quality [18]. One possible way to increase light efficiency in PFAL is extending the duration of lighting period. The use of long photoperiods (up to 24 h a day—continuous light, CL) is the pinnacle utilization of lighting as it provides continuous, 24 h of radiation to the crop. Theoretically, the use of CL provides constant energy for carbon assimilation, meaning larger biomass accumulation and yield [19]. Furthermore, the implementation of a supplemental CL strategy can have a positive economic implication as well [18] if no photo-injury occurs. However, the use of CL on plant cultivation presents a challenge, because plant responses are contradictory and are far from being fully understood. Past studies with conventional light sources have shown that long photoperiods of lighting cause photo-injury – leaf chlorosis, and reduction in leaf photosynthesis and yield in vegetable crops such as tomatoes, eggplant, cucumbers, and peppers, which have limited the application of this economically favorable lighting strategy in greenhouse vegetable production [20,21,22]. Recent research has shown that CL delivered by LEDs of different intensities and spectral quality can be used to improve yield and nutritional quality of lettuce plants by reducing nitrate content and enhancing phytochemical concentrations and antioxidant capacity [23,24,25,26].

Although previous reports have indicated that light intensity or light quality had an effect on the growth of microgreens, to our knowledge, little work has been published on the interaction between photoperiod, light intensity, and light quality on the growth and nutrient content of microgreens. This study investigated the physiological, growth, and yield response of four genotypes of *Brassicaceae* microgreens (arugula, broccoli, mizuna, and radish) to CL with different spectral quality (LED and FLU) under fully controlled growth conditions. Our aims were to test if CL, in combination with specific light spectra, could increase yield and nutritional value of the microgreens with respect to a 16 h photoperiod and determine if the response is dependent on light spectrum.

## 2. Results

### 2.1. Plant Growth and Yield

CL over a 9-day cycle (from day 4 after sowing to harvest), with two different light spectra (FLU and LED) and at 270 µmol m^−2^ s^−1^ of PAR neither impaired growth of four microgreens nor caused visual photodamage to the leaves. The growth and productivity of arugula, broccoli, mizuna, and radish plants were significantly affected by CL (Table 1). CL-grown plants outperformed plants grown under 16 h photoperiod in terms of production of fresh weight (FW) and dry weight (DW), except for mizuna in FLU-CL treatment. Plants in both CL treatments had much higher leaf mass per area (LMA). CL increased the growth and productive performances of plants with minor effects depending on the light quality applied.

Plants under CL were shorter, and although, not always, the differences between 16 h photoperiods and CL were significant (Table 1). The more pronounced decrease in hypocotyl length (HL) was observed in FLU treatments.

**Figure 1:**
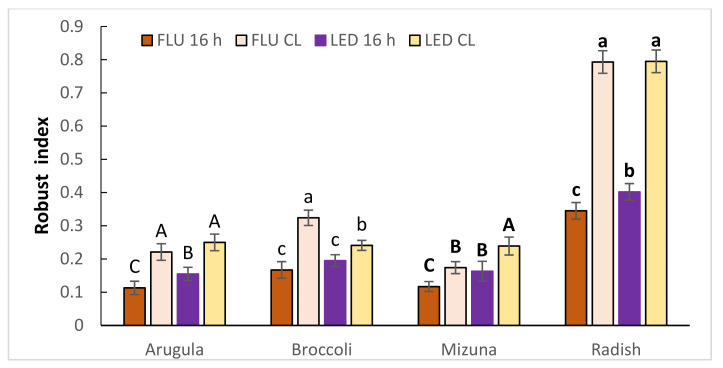
The robust index of arugula, broccoli, mizuna and radish microgreens grown under 16 h photoperiod or CL provided by fluorescent (FLU) or LED lamps. The results are presented as the mean ± standard error. Different letters for each plant species indicate significant differences between the mean values at *p* < 0.05.

Robust index (RI) was significantly higher in all CL-treated plants both in FLU and LED treatments (Figure 1). There was no effect of treatments on the hypocotyl diameter (HD).

Water content (WC) of microgreens was not significantly influenced by CL (Table 1), with the exception of LED-CL-treated arugula and radish plants, which was 9 and 7% lower WC compared to control plants grown under 16 h photoperiod, respectively.

The length of the first true leaf (LL) was higher in all microgreen plants treated by CL regardless of light quality, and effect was the greatest (up to 2–3-fold) in broccoli and radish (Table 1).

### 2.2. Photosynthetic Pigments

**Figure 2:**
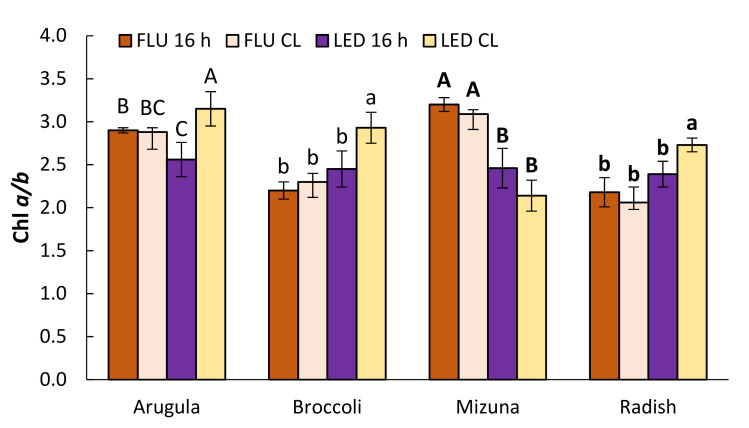
Chl *a/b* ratio of arugula, broccoli, mizuna, and radish microgreens grown under 16 h photoperiod or CL provided by fluorescent (FLU) or LED lamps. The results are presented as the mean ± standard error. Different letters for each parameter and plant species indicate significant differences between the mean values at *p* < 0.05.

**Figure 3:**
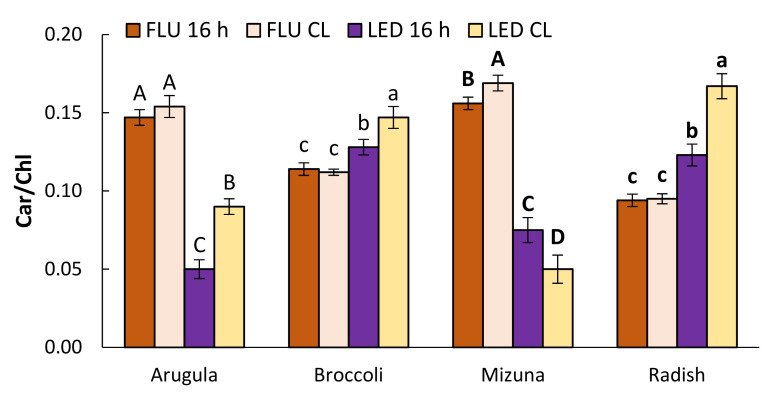
The ratio of carotenoid to chlorophyll content of arugula, broccoli, mizuna, and radish microgreens grown under 16 h photoperiod or CL provided by fluorescent (FLI) or LED lamps. The results are presented as the mean ± standard error. Different letters for each parameter and plant species indicate significant differences between the mean values at *p* < 0.05.

CL decreased total chlorophyll (Chl) content in arugula and mizuna, but not in broccoli and radish (Table 2). Arugula and mizuna plants in FLU treatments were more sensitive and lost more Chl compared to LED-illuminated plants. Similar trend was observed for carotenoid (Car) content. Higher levels of Chl *a/b* ratio (Figure 2) and Car/Chl ratio (Figure 3) were observed for LED-CL treatments.

### 2.3. Anthocyanins and Flavonoids

In all, CL-treated microgreens anthocyanins and flavonoids contents were significantly increased (Table 2), except for FLU-CL radish plants. The highest level of anthocyanins and flavonoids contents in arugula and broccoli were detected in LED-CL plants. Higher anthocyanins and other flavonoids contents were correlated with visible purple and blue coloration of abaxial leaf sell layers. 

### 2.4. Leaf Oxidative Stress and Antioxidative Enzyme Activity

The results indicate that all microgreens grown under CL had higher H_2_O_2_ content compared to control plants. In arugula, a 54% increase in H_2_O_2_ content was observed only in LED-CL plants, while mizuna had 2.5 times and 25% higher H_2_O_2_ content in FLU-CL and LED-CL treatment, correspondingly.

There were some differences in plant response induced by CL between microgreen species in respect to the level of lipid peroxidation determined in terms of malondyaldehyd (MDA) content and antioxidant enzyme activities. In arugula and broccoli, CL significantly increased the lipid peroxidation (Table 3) and effects were stronger in LED treatments. In mizuna and radish, CL treatments did not increase MDA content under neither of the two light qualities. The activities of antioxidant enzymes—catalase (CAT), superoxide dismutase (SOD), ascorbate peroxidase (APX), and guaiacol peroxidase (GPX) were increased by CL treatments to different extent, but the most dramatic increases were recorded for SOD activity in CL-grown broccoli and mizuna plants.

CL did not induce significant increase in the membrane permeability estimated through a relative electrolyte leakage (REL) from the cotyledon tissues in all plant species (Table 3). 

Higher proline content was observed in CL-treated plants (Table 3), except for FLU-CL-treated mizuna plants. 

## 3. Discussion

### 3.1. Plant Growth and Yield

In our research, FW, DW, and LMA of all studied species increased in CL-treated plants compared to plants grown under 16 h photoperiod. This plant response was rather expected as the CL treatments with the higher DLI provided additional light for photosynthetic activity and therefore biomass accumulation. In general, plant DM increases as DLI increases up to a light saturation point [27]. The higher productivity and the implied photosynthetic assimilation rate, measured in our CL treatments, support the hypothesis [19] that the use of CL provides constant energy for carbon assimilation, meaning larger biomass accumulation and yield if no CL-injury occurs. Obtained results show that all four plant species did not develop any symptoms of CL-induced injuries and therefore accumulated more dry mass. Samuoliene et al. [28] also reported similar increases in DW of tatsoi and red pak choi microgreens, as DLI increased. As for the sensitivity of studied plant species to CL, the only available report informs that the efficiency of rocket indoor cultivation was ameliorated by CL without detrimental effects on the yield and quality of edible leaves [18]. A major goal of commercial microgreen producers is to provide growth conditions that maximize as microgreens are sold on a FW basis [29]. In our study, this was the case with all four species in all CL treatments. RI relates individual components of hypocotyl volume (i.e., length and diameter) to DW and serves as a proxy for plant robustness. CL increased RI in all four microgreen species regardless of light spectrum due to decreased HL and increased DW. CL significantly accelerated the emergence of the first true leaf. As microgreens are typically harvested at the stage of the first true leaf, CL may serve as a way to reduce the time to harvest. There are numerous reports reviewed by Sysoeva et al. [20] testifying that CL increases developmental rate of plants. Thus, CL can increase yield and/or shorten production time in arugula, broccoli, mizuna, and radish microgreens.

### 3.2. Photosynthetic Pigments

The effects of light on photosynthetic pigment content varies depending on photoperiod [18,20,30,31], the light spectrum applied during plant growth [13,16,32], and plant species [20]. In this study chlorophyll content of broccoli and radish was not significantly influenced by photoperiod (or DLI) or light quality (Table 2). However, Chl content decreased in arugula and mizuna, which was not observed visually. This is important as Chl content is closely associated with human perception of the green pigmentation of leaves [33]. Total Chl content was also negatively affected when CL provided by RB LEDs was applied to rocket leaves [18]. In all LED-CL plants, we observed a reduction in LHCII, and an elevation of the Chl *a/b* ratio (Figure 2) and the Car/Chl ratio (Figure 3). Decrease in total Chl content in the leaves reduces the light absorption efficiency by photosynthetic apparatus per unit of leaf area and may serve as one of the protection mechanisms against excessive light. An increase in the Chl *a*/*b* ratio suggests that individual PSII complexes developed smaller LHCII during CL. It is considered as characteristic of plant adaptation to high light. The observed an increase in the ratio of yellow to green pigments under CL, which indicates a relatively higher concentration of Car in the pool of photosynthetic pigments, is also associated with their protective function during adaptation to excess lighting [34] as Car play a protective role against ROS as they are known to be very efficient physical and chemical quenchers of singlet oxygen and potent scavengers of other free radicals [35]. Thus, plants under CL either did not have less Chl than plants grown under 16 h photoperiod, or total Chl decrease was not that dramatic to decrease overall visual quality of microgreens. At the same time, increased Car content in arugula and radish adds nutritional value to microgreens as Car possess antioxidant properties that are beneficial for human health [36,37].

### 3.3. Leaf Oxidative Stress and Antioxidants

The increased MDA and H_2_O_2_ content in CL-treated plants suggest that CL increased photo-oxidative pressure in plants. In response to stress, plants accumulate antioxidant bioactive compounds. In this study, we recorded the increase in proline, anthocyanin, and flavonoid contents, and elevated activities of antioxidant enzymes (CAT, SOD, APX, and GPX). Higher concentrations of superoxide anion and the H_2_O_2_ concentrations as well as higher activity of CAT, SOD, and APX were also observed in CL-treated tomato plants compared to plants grown under 16 h photoperiod with the same DLI [38]. Increased content of anthocyanins was also reported for rocket leaves grown under CL provided by cool white LEDs, and by red and blue (RB) LED conditions, with the value doubling in the presence of RB radiation [18]. The role of antioxidants in scavenging free radicals suggests that they serve as essential compounds for human health, protecting the human organism against ROS [39]. Other results [27,40,41,42,43,44,45,46] demonstrate that higher DLI is normally beneficial to growth and nutritional quality of leafy plants. The question arises whether the higher DLI or extended photoperiod itself is a reason of the excess of absorbed light that increases photooxidative pressure and makes plant to produce photoprotective antioxidants. Thus, it was shown that plants may develop CL-induced injury even if DLI is not higher than normally required by plants under shorter photoperiods [47].

Horticultural *Brassicaceae* plants are valuable source of vitamins, minerals, antioxidants, and dietary fibers [48,49,50]. In addition, they can be cultivated as microgreens under such lighting conditions that enhance synthesis of antioxidants. The popularity of microgreens as a ‘functional’ food is gained due to high nutritional quality, and as a culinary ingredient due to their intense color, flavor, and tender texture [39,51]. Thus, CL provided by LEDs may add nutritional value to microgreens by increasing the antioxidant properties. These results are consistent with several studies that have reported the benefits of LEDs for nutritional value of horticultural crops reviewed by Loi et al. [13]. Moreover, low nitrate levels can add value to microgreens in the marketplace. The nitrate content is potentially harmful to human health. It was significantly reduced in the rocket leaves grown under CL conditions regardless of the light spectrum applied [18]. However, higher light intensity had little impact on nitrate levels in kale, cabbage and arugula microgreens [52], and mizuna [53]. Other studies have revealed that nitrate levels in lettuce and some microgreens were unaffected by LEDs when varying blue light proportion [43,54,55]. These and other findings indicated that additional environmental factors influenced nitrate accumulation [52]. Therefore, more research is required to address the relationship between species genotype, nitrogen supply and uptake, and light conditions, in order to produce microgreens with the lowest nitrate content.

## 4. Materials and Methods

### 4.1. Plant Material and Growth Conditions

Arugula (*Eruca vesicaria* subsp. *sativa* (Mill.) Thell.), broccoli (*Brassica oleracea* var. *italica* Plenck), mizuna *(Brassica rapa.* var. *nipposinica* (L.H.Bailey) Hanelt), and radish (*Raphanus sativus* var. *radicula* Pers.) were grown under controlled environmental conditions in a growth chamber (Snijders, The Netherlands). The average air temperature and relative air humidity were 22 ± 1 °C and 60 ± 5%, respectively. No supplemental CO_2_ was used in the experiment.

Trays (10 cm^2^) with coconut coir mats without drainage holes were used for microgreen culture. Eight trays were used for each species by sowing 1.8, 3.6, 3.6, and 4.8 g of arugula, broccoli, mizuna, and radish seeds evenly onto each mat. For the first 3 d after sowing trays of microgreens were placed to germinate in darkness and were top-irrigated with water. Once cotyledons were fully reflexed on the 4th day after sowing, a half-strength Hoagland’s nutrient solution [56] (pH 6.2−6.4) was added to each tray daily until harvest.

### 4.2. Light Treatments

Four light treatments were set from the 4th day after sowing until harvest (Table 4): (1) FLU-16h with a photoperiod of 16 h light/8 h dark provided by fluorescent lamps (F36W/T8 BRITEGRO, Sylvania, Germany) served as a control for fluorescent light treatment; (2) LED-16h with a photoperiod of 16 h light/8 h dark provided by LEDs (LED GL V300, China) served as a control for LED treatment; (3) FLU-CL with a photoperiod of 24 h continuous light provided by fluorescent lamps; (4) LED-CL with a photoperiod of 24 h continuous light provided by LEDs. LED light ratio (%) of red:green:blue was 50.3:21.1:17.6.

The PPFD of 270 µmol m^−2^ s ^−1^ was used in all treatments providing daily cumulative intensity of photosynthetically active quanta (as daylight integral, DLI) 15.6 mol m^−2^ day^−1^ and 23.3 mol m^−2^ day^−1^ for plants grown under 16 h photoperiod and CL, respectively. The PPFD was measured using LI-250A Light Meter (Li-COR Biosciences, Lincoln, NE, USA). All trays were systematically rearranged every day to minimize disproportion in light distribution.

### 4.3. Growth Measurements 

Microgreens were harvested at the visual appearance of true leaves, resulting in the harvest date of 12 days after sowing.

Ten seedlings of each species were randomly selected and measured to determine hypocotyl length (HL), first true leaf length (LL), fresh weight (FW), and dry weight (DW) for each light treatment. HL was measured from the base of the hypocotyl to the shoot apical meristem. To determine FW seedlings were weighed as quickly as possible to limit the losses through evaporation and then samples were dried at 105 °C in an oven until a constant dry weight (DW) was observed. FW and DW data were then used to report water content (WC, %). The values of leaf mass per area (LMA) were calculated as a ratio of a dry mass of the lamina discs to their area. Eight discs were cut from cotyledons with a 4-mm in diameter cork borer. The dry weight of the discs was determined after their drying to a constant weight at 105 °C.

Robust index (RI) = Hypocotyl diameter (HD)/Hypocotyl length (HL) × Dry weight (DW) [57]

### 4.4. Photosynthetic Pigment Content Measurement

Five plants per treatment were randomly selected for the following measurements. Content of Chl *a* and *b* and carotenoids (Car) was measured in 96% ethanol extracts with a SF2000 spectrophotometer (Spectrum, Russia) and calculated according to the known formulas [58]. The percentage of Chl in light harvesting complex II (LHCII) was calculated by accepting that almost all Chl *b* is in LHCII, and that the ratio of Chl *a* and *b* in LHCII is 1.2 [59].

### 4.5. Measurement of Lipid Peroxidation Levels 

The content of MDA, the end product of lipid peroxidation, was determined with a standard method based on the reaction of these substances with thiobarbituric acid (TBA) that produces a trimethine complex [60]. Leaf sample (0.1 g) was homogenized in 2 mL of 50 mM phosphate buffer (pH 7.0). The homogenate was centrifuged at 15,000× *g* for 15 min. To 1.0 mL aliquot of the supernatant, 2.0 mL of 0.5% TBA in 20% trichloroacetic acid was added. The mixture was heated at 95 °C for 30 min on the water bath and then cooled in an ice bath. After centrifugation at 10,000× *g* for 10 min the absorbance of the supernatant was recorded at 532 nm. The value for nonspecific absorption of each sample at 600 nm was also recorded and subtracted from the absorbance recorded at 532 nm. The concentration of MDA was calculated using an extinction coefficient of 155 mM^−1^ cm^−1^. The lipid peroxidation levels were expressed as micromoles of MDA per gram of FW.

### 4.6. Antioxidative Enzyme Activity Assays

For protein and antioxidant enzyme assays, leaf tissues (0.1 g) were ground in 4 mL of 50 mM potassium phosphate buffer (pH 7.8) using a chilled pestle and mortar. The extraction buffer contained 0.1 mM EDTA and 1% (*w*/*v*) polyvinylpyrrolidone. The homogenate was centrifuged at 14,000× *g* for 20 min at 4 °C, and the supernatants thus collected were used for the assays of catalase (CAT, EC 1.11.1.6), superoxide dismutase (SOD, EC 1.15.1.1), ascorbate peroxidase (APX, EC 1.11.1.11), and guaiacol peroxidase (GPX, EC 1.11.1.7), and protein determinations. 

CAT activity was determined using spectrophotometer SF-2000 (OKB Spectr, Russia) by measuring the rate of H_2_O_2_ disappearance at 240 nm [61]. The reaction mixture contained 50 mM potassium phosphate buffer (pH 7.0) and 10.5 mM H_2_O_2_. The reaction was run at 25 °C for 1 min, after adding the enzyme extract and rate of decrease in absorbance at 240 nm (E  =  39.4 mM^−1^cm^−1^) was used to calculate the enzyme activity. CAT activity was expressed in µmol H_2_O_2_ per minute per mg of protein. 

SOD activity assay was based on the measurement of inhibition in the photochemical reduction in nitroblue tetrazolium (NBT) spectrophotometrically. The reaction mixture contained 50 mM K-phosphate buffer (pH 7.8), 13 mM methionine, 75 μM NBT, 0.1 μM EDTA, 4 μM riboflavin, and the required amount of enzyme extract. The reaction was triggered by adding riboflavin and placing the tubes under fluorescent lamps for 30 min. A complete reaction mixture without enzyme served as control. A non-irradiated complete reaction mixture served as a blank. One unit of SOD activity was defined as the amount of enzyme required to cause 50% inhibition of the reduction in NBT as monitored at 560 nm according to [62].

APX was assayed by the method described by Nakano, Asada [63]. The reaction mixture contained 50 mM potassium phosphate buffer (pH 7.0), 0.2 mM EDTA, 0.5 mM ascorbic acid, and 0.25 mM H_2_O_2_. The reaction was triggered by the addition of H_2_O_2_ after adding the enzyme extract. The decrease in absorbance at 290 nm for 1 min was recorded and the amount of APX was calculated from the extinction coefficient 2.8 mM^−1^cm^−1^.

The activity of GPX was determined spectrophotometrically by measuring the increase in absorbance at 470 nm [64]. The reaction mixture contained 80 mmol/L guaiacol and 10 mmol/L H_2_O_2_ in 0.066 mol/L phosphate buffer (pH = 7.4). The enzymatic reaction was started by adding 0.05 mL of the extract to 2 mL of reaction mixture. The SOD, APX, and GPX activity values are expressed as unit U mg^−1^ protein.

The concentration of protein was determined according to Bradford [65] using bovine serum albumin (BSA) (Dia-M, Russia) as a standard.

### 4.7. Measurement of Relative Electrolyte Leakage (REL)

REL from the cotyledon tissues was determined to estimate the membrane permeability. Ten 4 mm in diameter leaf discs were rinsed with distilled water, blotted with filter paper, and placed into 10 mL test tubes with distilled water. After 2 h shaking at 23 °C, the electric conductivity of the solution (E1) was measured by a conductometer (Ekspert-002, Ekoniks-Ekspert, Russia) at the same temperature. Then, the test tubes were heated until boiling, cooled to room temperature, and the full electrolyte leakage (E2) was evaluated. REL was calculated as a percentage of the full leakage by the formula REL = 100 × E1/E2%.

### 4.8. Hydrogen Peroxide Content

Hydrogen peroxide content was determined according to Velikova et al. [66]. Leaf tissues (0.1 g) were homogenized in ice bath with 5 mL 0.1% (*w*/*v*) TCA. The homogenate was centrifuged at 12,000× *g* for 15 min at 4 °C and 0.5 mL of supernatant was added to 0.5 mL potassium phosphate buffer (pH 7.0) and 1 mL 1M KI. The absorbance of supernatant was measured at 390 nm. The content of H_2_O_2_ was calculated by comparison with a standard calibration curve.

### 4.9. Proline

Free proline content in the leaf tissues was estimated according to Bates et al. [67]. Fresh leaf samples (0.3 g) were homogenized in 6 mL of 3% sulfosalicylic acid and the homogenate was centrifuged at 5100× *g* for 5 min. Then, 2 mL of the supernatant was mixed with 2 mL freshly prepared ninhydrin reagent (0.5 g ninhydrin in 50 mL of 60% acetic acid). The colour reaction developed after incubation of the samples for 1 h in a boiling water bath. After warming to 25 °C, the absorbance was measured at 520 nm. The concentration of proline was estimated by referring to a standard curve of L-proline and expressed in µmol g^−1^ FW.

### 4.10. Anthocyanins and Flavonoids

Anthocyanins were extracted from leaves according to Kang et al. [68]. Fresh leaves tissues (0.1 g) were homogenized in 2 mL of 95% ethanol-1.5 N HCl- (85:15, *v*:*v*). After overnight extraction at 4 °C in darkness, each sample was centrifuged at 10,000× *g* for 5 min. The absorbance of the supernatant was measured at 533 nm (peak of absorption of anthocyanin) and 657 nm (peak of absorption of Chl degradation products). The results were plotted as a difference in absorption at 530 and 657 nm relative to tissue fresh weight (∆A∙g^−1^ FW) and the formula ∆A = A530 − 1/4A657 was used to deduct the absorbance contributed by chlorophyll and its degradation products in the extract [69].

The relative amounts of flavonoids were measured spectrophotometrically [70,71,72]. The supernatant for anthocyanins was diluted 10 times and the absorbance was measured at 300 nm. Flavonoids content in the sample was expressed as absorbance at 530 nm g^−1^ fresh weight of tissue.

### 4.11. Data Analysis

The experiment was performed twice overtime for each species and data were pooled across replications. The figures show mean values and standard errors. Significant differences between the means were revealed at *p* < 0.05 using one-way ANOVA analysis (the least significant difference test).

## 5. Conclusions

This study investigated the responses of four microgreen genotypes to CL provided by florescent lights and RB LEDs with PPFD of 270 µmol m^−2^ s^−1^. Combining results in growth, yield, and nutritional quality of arugula, broccoli, mizuna, and radish microgreens, we suggest that CL had a significant impact on microgreen growth and nutritional quality. In most cases effects were more pronounced under LED lighting. LED-CL resulted in higher yield and RI compared to shorter photoperiod, and led to increased content of phytochemicals with antioxidative properties (carotenoids, anthocyanins, flavonoids, proline, and antioxidant enzymes). This added nutritional value to microgreens that are used as functional foods providing health benefits.

## Figures and Tables

**Table 1:** Growth characteristics of microgreens grown under 16 h photoperiod or continuous light provided by fluorescent (FLU) lamps or LEDs.

Parameter	Arugula				Broccoli				Mizuna				Radish			
	FLU		LED		FLU		LED		FLU		LED		FLU		LED	
	16 h	CL	16 h	CL	16 h	CL	16 h	CL	16 h	CL	16 h	CL	16 h	CL	16 h	CL
HL, mm	35.5 ± 1.0 A	31.7 ± 1.0 B	36.8 ± 1.2 A	35.2 ± 0.9 A	36.0 ± 1.5 b	34.0 ± 1.7 b	51.2 ± 2.2 a	49.7 ± 1.6 a	46.0 ± 0.8 **A**	35.7 ± 1.4 **B**	47.3 ± 1.7 **A**	38.5 ± 1.7 **B**	37.7 ± 1.7 **c**	32.8 ± 0.9 **d**	52.3 ± 2.4 **a**	47.4 ± 2.4 **b**
LL, mm	2.8 ± 0.5 D	4.2 ± 0.2 C	16.5 ± 0.8 B	19.2 ± 1.4 A	2.2 ± 0.4 b	6.8 ± 0.5 a	2.5 ± 0.7 b	5.7 ± 0.7 a	12.8 ± 0.6 **B**	14.5 ± 1.3 **A**	5.4 ± 0.5 **D**	6.9 ± 0.6 **C**	6.2 ± 0.6 **b**	11.3 ± 2.6 **a**	5.5 ± 0.7 **b**	12.5 ± 0.7 **a**
FW, g	0.041 ± 0.003 C	0.052 ± 0.003 B	0.052 ± 0.003 B	0.060 ± 0.004 A	0.058 ± 0.008 b	0.096 ± 0.005 a	0.070 ± 0.010 b	0.090 ± 0.010 a	0.062 ± 0.004 **C**	0.054 ± 0.003 **C**	0.076 ± 0.003 **B**	0.088 ± 0.004 **A**	0.162 ± 0.006 **d**	0.268 ± 0.009 **b**	0.230 ± 0.020 **c**	0.300 ± 0.010 **a**
DW, mg	4.0 ± 0.4 D	7.0 ± 0.6 B	5.7 ± 0.5 C	8.8 ± 0.4 A	6.0 ± 1.0 c	11.0 ± 1.0 a	10.0 ± 1.0 b	12.0 ± 1.0 a	5.4 ± 0.8 **D**	6.2 ± 0.7 **C**	7.7 ± 0.5 **B**	9.2 ± 0.7 **A**	13.0 ± 1.0 **d**	26.0 ± 3.0 **b**	21.0 ± 2.6 **c**	38.0 ± 2.0 **a**
LMA, mg/cm^2^	2.2 ± 0.2 C	3.8 ± 0.5 B	2.5 ± 0.4 C	5.7 ± 0.4 A	3.3 ± 0.3 c	4.4 ± 0.6 b	5.2 ± 0.4 a	5.6 ± 0.6 a	2.4 ± 0.2 **C**	3.4 ± 0.5 **B**	3.3 ± 0.2 **B**	3.9 ± 0.3 **A**	2.8 ± 0.3 **b**	2.9 ± 0.1 **b**	2.9 ± 0.3 **b**	4.9 ± 0.5 **a**
WC, %	90.2 ± 0.5 A	86.6 ± 0.5 B	88.5 ± 1.5 AB	80.6 ± 1.4 C	90.4 ± 0.6 a	88.9 ± 0.5 a	83.8 ± 1.3 b	82.2 ± 1.3 b	91.2 ± 0.4 **A**	88.6 ± 1.2 **B**	87.9 ± 0.9 **B**	86.2 ± 0.7 **C**	91.6 ± 0.5 **a**	90.3 ± 1.1 **a**	92.1 ± 2.2 **a**	85.6 ± 1.6 **b**

HL—hypocotyl length, LL—first true leaf length, FW—fresh weight, DW—dry weight, LMA—leaf mass per area. WC—water content. Different letters for each plant species indicate significant differences between the mean values at *p* < 0.05.

**Table 2:** Leaf chlorophyll (*a + b)* content, share of chlorophyll in light-harvesting complex II (LHCII), carotenoid, anthocyanin, and flavonoid contents of microgreens grown under 16 h photoperiod or continuous light provided by fluorescent (FLU) lamps or LEDs.

Parameter	Arugula				Broccoli				Mizuna				Radish			
	FLU		LED		FLU		LED		FLU		LED		FLU		LED	
	16 h	CL	16 h	CL	16 h	CL	16 h	CL	16 h	CL	16 h	CL	16 h	CL	16 h	CL
Chl *a + b,* mg/mg DW	12.9 ± 0.1 A	6.8 ± 0.2 C	12.3 ± 0.7 A	9.6 ± 0.4 B	6.6 ± 0.7 b	7.2 ± 0.3 b	9.0 ± 0.3 a	8.1 ± 0.6 ab	8.8 ± 1.1 **A**	6.3 ± 0.3 **B**	5.4 ± 0.3 **C**	4.4 ± 0.2 **D**	7.3 ± 0.5 **a**	7.8 ± 0.5 **a**	6.5 ± 0.2 **b**	6.2 ± 0.3 **b**
LHCII, %	56.5 ± 0.5 B	56.9 ± 0.8 B	63.6 ± 3.6 A	54.6 ± 3.4 B	68.8 ± 2.1 a	68.5 ± 3.6 a	65.9 ± 4.3 a	59.6 ± 3.8 a	52.6 ± 1.0 **B**	53.9 ± 0.71 **B**	65.7 ± 3.8 **A**	70.8 ± 2.5 **A**	71.3 ± 4.4 **a**	74.1 ± 4.4 **a**	66.1 ± 3.1 **b**	59.1 ± 2.7 **b**
Carote-noids, mg/mg DW	1.9 ± 0.1 A	1.1 ± 0.1 B	0.7 ± 0.1 C	0.9 ± 0.1 B	0.8 ± 0.1 c	0.9 ± 0.1 b	1.2 ± 0.1 a	1.3 ± 0.1 a	1.4 ± 0,1 **A**	1.1 ± 0.1 **B**	0,4 ± 0.1 **C**	0,2 ± 0.1 **D**	0.8 ± 0.1 **b**	0.9 ± 0.1 **a**	0.8 ± 0.1 **b**	1.0 ± 0.1 **a**
Anthocy-anins, mg/mg FW	1.4 ± 0.1 C	2.0 ± 0.5 B	1.7 ± 0.2 B	5.7 ± 0.2 A	1.5 ± 0.2 b	2.6 ± 0.6 a	1.3 ± 0.5 b	2.5 ± 0.2 a	0.3 ± 0.1 **B**	0.8 ± 0.3 **A**	0.3 ± 0.0 **B**	0.5 ± 0.1 **A**	0.2 ± 0.0 **b**	0.4 ± 0.0 **a**	0.2 ± 0.0 **b**	0.4 ± 0.0 **a**
Flavo-noids, mg/mg FW	15.1 ± 0.9 D	18.7 ± 1.9 C	30.7 ± 2.1 B	41.9 ± 1.8 A	25.5 ± 0.7 ab	28.3 ± 2.3 a	23.0 ± 1.2 b	31.4 ± 1.9 a	20.6 ± 1.1 **C**	27.4 ± 2.7 **A**	18.9 ± 1.4 **C**	23.6 ± 0.9 **B**	31.4 ± 2.0 **a**	31.1 ± 1.1 **a**	23.9 ± 2.2 **b**	32.6 ± 2.5 **a**

Different letters for each plant species indicate significant differences between the mean values at *p* < 0.05.

**Table 3:** The malondialdehyde (MDA), H_2_O_2_, proline content, relative electrolyte leakage (REL) and catalase (CAT), superoxide dismutase (SOD), ascorbate peroxidase (APX), and guaiacol peroxidase (GPX) enzyme activities of microgreens grown under 16 h photoperiod or continuous light provided by fluorescent (FLU) lamps or LEDs.

Parameter	Arugula				Broccoli				Mizuna				Radish			
	FLU		LED		FLU		LED		FLU		LED		FLU		LED	
	16 h	CL	16 h	CL	16 h	CL	16 h	CL	16 h	CL	16 h	CL	16 h	CL	16 h	CL
MDA, µmol/g FW	43.2 ± 4.6 C	92.4 ± 5.5 A	20.1 ± 0.7 D	55.5 ± 4.2 B	26.5 ± 2.4 b	47.9 ± 10.5 a	18.4 ± 1.3 c	60.6 ± 7.6 a	14.2 ± 1.2 **A**	14.2 ± 1.2 **A**	11.9 ± 0.5 **A**	13.0 ± 1.1 **A**	21.8 ± 2.0 **a**	20.6 ± 1.0 **a**	18.4 ± 1.3 **b**	18.0 ± 0.5 **b**
H_2_O_2_, µmol/g FW	0.93 ± 0.06 A	1.00 ± 0.22 A	0.54 ± 0.04 C	0.83 ± 0.07 B	0.61 ± 0.12 b	0.87 ± 0.04 a	0.71 ± 0.05 b	0.93 ± 0.02 a	0.70 ± 0.09 **B**	1.74 ± 0.13 **A**	0.63 ± 0.04 **C**	0.79 ± 0.04 **B**	0.68 ± 0.02 **b**	0.84 ± 0.02 **a**	0.40 ± 0.05 **d**	0.55 ± 0.04 **c**
REL, %	8.7 ± 0.6 C	9.8 ± 0.8 C	17.5 ± 2.0 A	14.1 ± 1.4 B	11.3 ± 0.3 a	12.4 ± 0.6 a	13.7 ± 1.8 a	12.2 ± 1.5 a	5.1 ± 0.4 **B**	4.6 ± 0.3 **B**	10.6 ± 0.2 **A**	8.9 ± 0.5 **A**	12.1 ± 0.6 **a**	9.9 ± 0.3 **b**	12.7 ± 0.5 **a**	12.7 ± 2.1 **a**
Proline, µmol/g FW	40.9 ± 1.1 C	49.4 ± 3.9 B	27.4 ± 2.5 D	182 ± 21 A	53.5 ± 4.3 a	58.4 ± 7.5 a	26.5 ± 2.8 c	36.5 ± 3.5 b	49.5 ± 4.3 **B**	60.2 ± 2.9 **A**	11.5 ± 0.3 **C**	12.5 ± 1.2 **C**	20.8 ± 1.5 **b**	58.4 ± 0.8 **a**	7.6 ± 1.3 **c**	17.1 ± 4.7 **b**
CAT, µmol/(mg protein min)	31.1 ± 3.0 C	42.2 ± 4.1 A	39.9 ± 3.2 B	47.0 ± 1.3 A	39.3 ± 2.1 b	55.4 ± 5.0 a	32.3 ± 2.6 b	36.4 ± 3.9 b	29.3 ± 2.4 **D**	42.4 ± 3.7 **B**	38.3 ± 5.5 **C**	49.0 ± 2.1 **A**	19.1 ± 1.7 **b**	20.3 ± 1.9 **b**	10.4 ± 2.8 **c**	30.1 ± 3.1 **a**
SOD, U/mg protein	32.1 ± 2.8 A	35.3 ± 2.6 A	5.0 ± 1.0 C	9.0 ± 1.0 B	5.6 ± 1.6 c	18.4 ± 1.5 a	6.6 ± 1.2 c	10.8 ± 0.7 b	41.0 ± 3.6 **B**	57.2 ± 6.5 **A**	13.0 ± 4.0 **C**	17.0 ± 3.0 **C**	7.4 ± 1.1 **b**	9.9 ± 0.6 **b**	15.3 ± 1.4 **a**	15.6 ± 1.9 **a**
APX, µmol/(mg protein min)	93.2 ± 7.9 B	125.0 ± 23.2 A	113.1 ± 16.3 B	149.3 ± 13.6 A	78.4 ± 6.1 b	121.3 ± 12.5 a	79.4 ± 13.3 b	131.3 ± 24.7 a	95.9 ± 16.3 **B**	231.0 ± 41.3 **A**	96.4 ± 23.7 **B**	114.2 ± 14.1 **B**	64.4 ± 4.4 **c**	65.4 ± 12.2 **c**	143.7 ± 2.9 **b**	163.2 ± 3.3 **a**
GPX, µmol/(mg protein min)	17.0 ± 2.8 A	18.3 ± 3.7 A	19.1 ± 2.7 A	20.3 ± 1.9 A	205.2 ± 18.2 b	256.3 ± 20.0 a	193.3 ± 31.4 b	225.7 ± 28.6 a	178.4 ± 21.0 **B**	348.4 ± 72.9 **A**	180.4 ± 20.3 **B**	235.8 ± 71.6 **A**	113.3 ± 3.9 **b**	126.2 ± 17.0 **ab**	141.4 ± 14.8 **a**	146.2 ± 17.5 **a**

Different letters for each plant species indicate significant differences between the mean values at *p* < 0.05.

**Table 4:** Light conditions (light source, photoperiod, light intensity, and daily light integral) of treatments.

Treatments	Light Source	Photoperiod, h	PAR, μmol m^−2^ s^−1^	DLI, mol m^−2^ day^−1^
FLU-16 h (control FLU)	FLU	16	270	15.6
LED-16 h (control LED)	LED	16	270	15.6
FLU-CL	FLU	24	270	23.3
LED-CL	LED	24	270	23.3
